# Transforming Patient Feedback Into Actionable Insights Through Natural Language Processing: Knowledge Discovery and Action Research Study

**DOI:** 10.2196/69699

**Published:** 2025-08-26

**Authors:** Ravi Shankar, Alexander Yip

**Affiliations:** 1 Medical Affairs – Research Innovation & Enterprise, Alexandra Hospital, National University Health System, 378 Alexandra Rd, Singapore, 159964, Singapore, 65 83797930; 2 Alexandra Research Centre for Healthcare in a Virtual Environment, Alexandra Hospital, National University Health System, Singapore, Singapore

**Keywords:** natural language processing, text mining, health care quality improvement, knowledge discovery in databases, action research, sentiment analysis, artificial intelligence, AI

## Abstract

**Background:**

Patient feedback has emerged as a critical measure of health care quality and a key driver of organizational performance. Traditional manual analysis of unstructured patient feedback presents significant challenges as data volumes grow, making it difficult to extract meaningful patterns and actionable insights efficiently.

**Objective:**

We attempted to develop and evaluate a comprehensive methodology for analyzing patient feedback data using natural language processing and Knowledge Discovery in Databases (KDD) approaches, aiming to identify key patterns, themes, and variations in patient experiences across different demographic groups and care settings, and to translate these insights into actionable improvements in health care delivery.

**Methods:**

This study applied an integrated KDD-action research framework to analyze 126,134 patient feedback entries collected at Alexandra Hospital, Singapore, in 2023. A comprehensive suite of text mining techniques, including sentiment analysis, topic modeling, emotion detection, and aspect-based sentiment analysis, was employed to uncover patterns in patient-reported experiences. The dataset included 92,578 (73.4%) entries containing free-text comments, comprising 1,568,932 tokens with an average comment length of 16.9 words. Multiple analytical techniques were used to ensure the validity and reliability of the findings. Stakeholder engagement throughout the research process facilitated the translation of analytical insights into practical improvements. The study was granted an exemption from ethical review by the National Healthcare Group Domain Specific Review Board (number: NUS-IRB-2025-087E), with a waiver of informed consent granted for this retrospective analysis of deidentified patient feedback data.

**Results:**

Text mining analysis revealed a moderately positive overall sentiment across the feedback corpus (average polarity score: 0.42), with 68.8% (63,685/92,578) of comments classified as positive, 25.4% (23,515/92,578) as neutral, and 5.8% (5378/92,578) as negative. Topic modeling identified 10 distinct topics, including staff attitude and service (9443/92,578, 10.2%), health care staff professionalism (9350/92,578, 10.1%), hospital environment (9258/92,578, 10.0%), and waiting time (9258/92,578, 10.0%). Aspect-based sentiment analysis highlighted nurse attitude (sentiment score: 0.65), staff helpfulness (0.61), and doctor expertise (0.58) as the most positive aspects, while waiting time (−0.42) and billing transparency (−0.28) emerged as the most negative. Demographic segmentation revealed significant variations in patient priorities, with younger patients (<35 years) expressing 37% more concerns about digital accessibility and efficiency than older patients who valued face-to-face interactions 42% more highly. Implementation of targeted interventions based on these findings resulted in measurable improvements, including an 18% increase in waiting time satisfaction, a 15% improvement in doctor-patient communication ratings, and a 23% reduction in billing-related complaints.

**Conclusions:**

The integration of natural language processing techniques with KDD and action research principles provides a powerful framework for transforming unstructured patient feedback into actionable insights for health care improvement. This approach enables health care organizations to understand the complex patterns and drivers of patient experience, identify targeted improvement opportunities, and implement evidence-based initiatives that enhance care quality and patient-centeredness.

## Introduction

In the rapidly evolving landscape of health care, patient experience has emerged as a critical measure of care quality and a key driver of organizational performance [1]. As health care systems shift toward patient-centeredness and value-based care, the ability to understand and enhance the entire patient journey has become a strategic imperative [2]. At the heart of this transformation lies the effective collection, analysis, and application of patient feedback data [3].

Traditionally, health care organizations have relied on structured surveys to assess patient experiences. While providing standardized metrics, surveys often fail to capture the full richness and complexity of patients’ perceptions and emotions [4]. Open-ended comments, where patients express their thoughts in their own words, offer valuable, complementary insights into satisfaction drivers [5]. However, the unstructured nature of free-text feedback presents significant challenges for traditional manual analysis, especially as data volumes grow [6]. The patterns and themes within may not be readily apparent, limiting the depth of insights [7].

Recent advancements in text mining and natural language processing (NLP) have opened new possibilities for automated analysis of free-text patient feedback [8]. Machine learning algorithms and computational linguistic methods allow for efficiently processing large volumes of unstructured data to extract meaningful knowledge [9]. Techniques like sentiment analysis, topic modeling, and aspect-based sentiment analysis have been successfully applied to patient experience data in various contexts, uncovering insights into factors shaping perceptions and behaviors [10-14].

Despite these advances, significant gaps remain in the literature regarding the systematic integration of multiple text mining techniques within established frameworks. Previous studies have typically employed single analytical methods (eg, only sentiment analysis or topic modeling), which can limit the depth and breadth of insights [9,15]. Moreover, most existing research focuses solely on the technical aspects of analysis without addressing the critical challenge of translating computational findings into practical health care improvements [16]. There is a particular lack of frameworks that combine rigorous data mining methodologies with stakeholder engagement processes to ensure that insights are both technically sound and practically actionable.

This study addresses these gaps by developing and evaluating a comprehensive framework that (1) integrates multiple NLP techniques within the Knowledge Discovery in Databases (KDD) process to provide multidimensional analysis of patient feedback; (2) incorporates action research principles to ensure stakeholder engagement throughout the analysis process; and (3) demonstrates how analytical insights can be systematically translated into measurable health care improvements.

The primary objective of this study is to develop and validate an integrated methodology that combines NLP, knowledge discovery, and action research principles to transform unstructured patient feedback into actionable insights for health care quality improvement.

## Methods

### Study Design and Theoretical Framework

**Figure 1.**
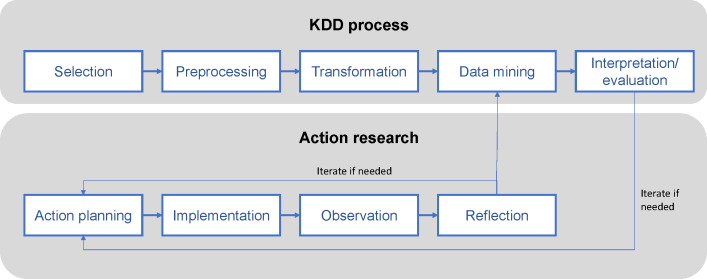
Research framework. KDD: Knowledge Discovery in Databases.

This study employed a mixed methods approach integrating the KDD process [17] with action research principles [18] to analyze patient feedback data. The KDD process provided a systematic framework for extracting patterns from large-scale unstructured data through 5 stages: selection, preprocessing, transformation, data mining, and interpretation/evaluation. Action research complemented this technical approach by ensuring stakeholder engagement and practical relevance through iterative cycles of planning, action, observation, and reflection [19]. This hybrid framework enabled both rigorous computational analysis and contextually grounded interpretation of the findings (Figure 1).

### Data Selection

The dataset consists of 126,134 patient feedback entries collected through the hospital’s patient experience portal in 2023, including structured metadata fields (eg, patient demographics and visit type) and unstructured free-text comments. The dataset was extracted and anonymized prior to analysis.

### Data Preprocessing

The free-text comment fields underwent systematic preprocessing using a standardized pipeline implemented in Python 3.8 with the Natural Language Toolkit (NLTK) library version 3.6 [20] (detailed data preprocessing steps are provided in Section S1 in Multimedia Appendix 1).

The preprocessed corpus retained 1,568,932 tokens from the original 92,578 comments, representing an average reduction of 23% in token count while preserving semantic content.

### Ethical Considerations

This study was reviewed by the National Healthcare Group Institutional Review Board and granted exemption (number: NUS-IRB-2025-087E) from full ethics review as it involved retrospective analysis of fully deidentified patient feedback data from routine hospital operations. All personally identifiable information was removed prior to analysis, including names, identification numbers, and contact details. Data were stored on secure, password-protected servers, with access restricted to authorized research personnel only.

### Data Transformation

The preprocessed text data were transformed using techniques like term frequency-inverse document frequency (TF-IDF), n-gram extraction, and part-of-speech (POS) tagging to prepare it for mining (detailed data transformation steps are provided in Section S2 in Multimedia Appendix 1)

Multiple complementary text mining techniques were applied to extract insights from different dimensions of the patient feedback data:

1. Sentiment analysis: TextBlob (version 0.15.3; Loria S) was used to calculate sentiment polarity scores ranging from −1 (most negative) to +1 (most positive). Comments were classified into positive (>0.1), neutral (−0.1 to 0.1), and negative (<−0.1) categories.
2. Topic modeling: Latent Dirichlet allocation was implemented using Gensim (version 4.1.2; Řehůřek R) with 10 topics, α=.01, and β=.01 parameters. Model performance was evaluated using coherence scores and perplexity metrics.
3. Aspect-based sentiment analysis: A hybrid approach combining rule-based aspect extraction with machine learning–based sentiment classification was developed. Aspects were identified using dependency parsing and predefined health care–specific patterns.
4. Emotion detection: The NRC Emotion Lexicon was used to detect 8 basic emotions (joy, trust, fear, surprise, sadness, anger, disgust, and anticipation) in patient comments.
5. Named entity recognition: A combination of spaCy’s pretrained models and custom health care entity patterns was used to identify mentions of health care professionals, departments, procedures, and facilities.

### Interpretation/Evaluation

The text mining and segmentation results were interpreted and evaluated collaboratively with key stakeholders, following the action research approach, including insight synthesis, stakeholder validation, recommendation development, action planning, implementation and monitoring, and reflection and learning.

Three stakeholder workshops were conducted with health care professionals (n=15), administrators (n=8), and patient representatives (n=5) to validate the findings and co-develop improvement strategies. Workshop discussions were recorded, transcribed, and analyzed thematically to ensure comprehensive integration of stakeholder perspectives.

## Results

### Data Selection and Preprocessing Results

The initial data selection and preprocessing resulted in a clean, structured dataset of 126,134 feedback entries, with 92,578 (73.4%) containing at least one free-text comment. The preprocessed corpus consisted of 1,568,932 tokens, with an average comment length of 16.9 words.

### Data Transformation Results

The transformation steps, including TF-IDF weighting, n-gram extraction, and POS tagging, generated rich features for the text mining analyses. The TF-IDF scores highlighted potentially significant terms like “nurse,” “doctor,” “care,” “staff,” “time,” and “wait.” N-gram analysis revealed frequent phrases, providing more context-specific insights into patient experience domains, such as “friendly staff,” “long waiting time,” “excellent care,” and “room condition.” POS tagging showed that nouns and adjectives were most common, indicating a focus on describing aspects, entities, and qualities of care experience. These transformed features enabled a more nuanced exploration of the feedback content and structure.

### Text Mining Results

Applying the suite of text mining techniques yielded multidimensional insights into the patient feedback data.

#### Word Cloud

Word cloud analysis provided an engaging visual overview of the most salient terms in the patient feedback corpus. The dominant words like “nurse,” “doctor,” “staff,” “care,” “time,” and “wait” indicated the centrality of human interactions, clinical processes, and environmental factors in shaping patient experiences (Multimedia Appendix 2).

#### N-Gram Analysis

**Figure 2.**
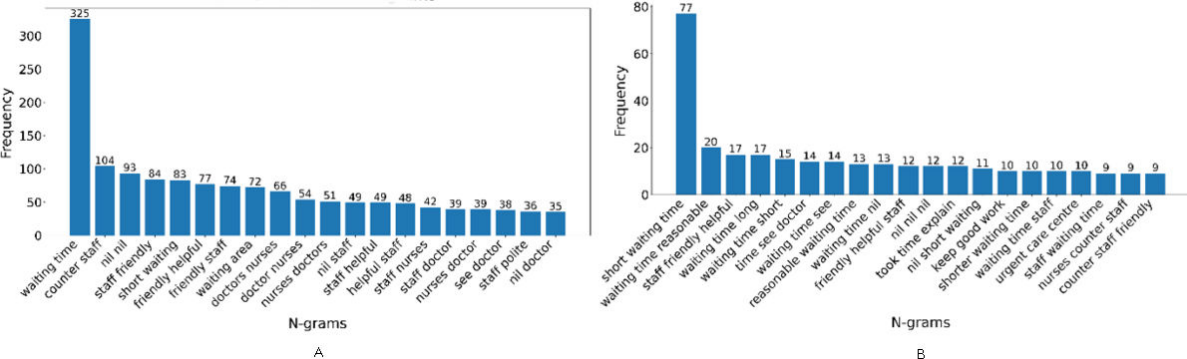
Top 20 bigrams (A) and trigrams (B) in patient feedback.

N-gram analysis uncovered the most frequent word sequences, offering a more granular view of the key themes and topics. The top bigrams and trigrams, such as “friendly staff,” “long waiting,” “good care,” “clean room,” and “discharge process,” revealed specific aspects frequently mentioned by patients, both positively and negatively, providing valuable context and nuance to the single-word insights from the word cloud (Figure 2).

#### Sentiment Analysis

**Figure 3.**
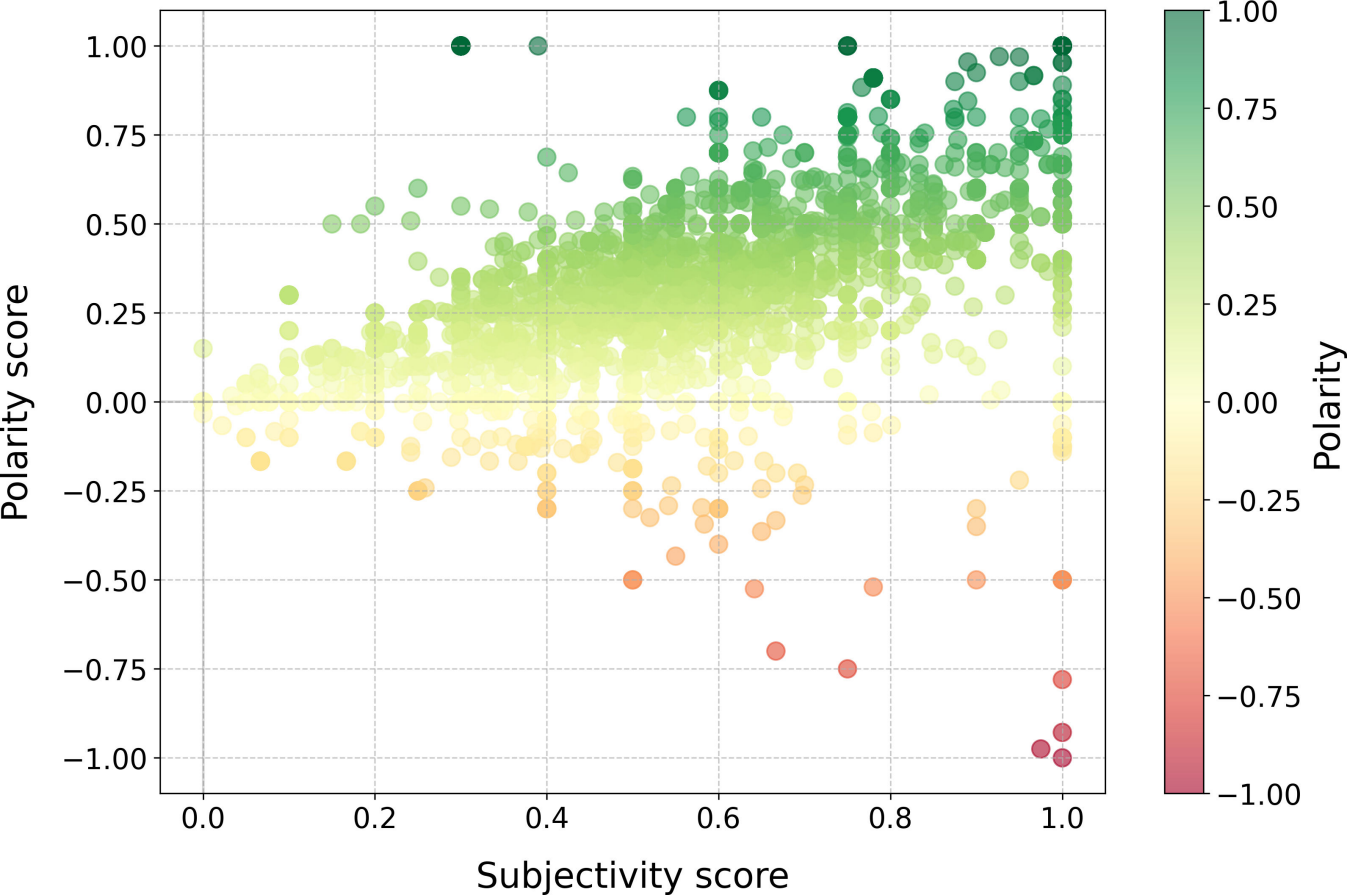
Sentiment analysis of patient feedback.

Sentiment analysis using the TextBlob model revealed a moderately positive overall sentiment across the feedback corpus, with an average polarity score of 0.42 (on a scale from −1 to +1). The distribution of sentiment categories showed that 68.8% (63,685/92,578) of comments were positive, 25.4% (23,515/92,578) were neutral, and 5.8% (5378/92,578) were negative. A more detailed analysis of sentiment polarity and subjectivity scores for individual comments revealed significant variation across different patient subgroups (Figure 3). This sentiment distribution underscores the importance of examining sentiment at a granular level to identify specific issues and opportunities for improvement, rather than relying solely on overall average scores.

#### Emotion Detection

Emotion detection using the EmoLex lexicon revealed a diverse range of emotions expressed in the patient comments. The most prevalent emotions were joy (30,180/92,578, 32.6%), trust (26,200/92,578, 28.3%), anticipation (22,311/92,578, 24.1%), and surprise (18,886/92,578, 20.4%), reflecting the generally positive sentiment. However, high proportions of comments also contained negative emotions, such as sadness (17,312/92,578, 18.7%), anger (14,072/92,578, 15.2%), fear (12,776/92,578, 13.8%), and disgust (8795/92,578, 9.5%), highlighting areas of patient dissatisfaction and concern (Multimedia Appendix 3).

#### Linguistic Inquiry and Word Count Analysis

Linguistic Inquiry and Word Count analysis provided a comprehensive set of linguistic and psychological metrics for the feedback comments. The comments had a relatively high percentage of personal pronouns (13,146/92,578, 14.2%), positive emotion words (4814/92,578, 5.2%), social words (11,480/92,578, 12.4%), and cognitive process words (9443/92,578, 10.2%), indicating a personal, subjective, interpersonal, and reflective narrative style. Health-related words were moderately frequent (4259/92,578, 4.6%). Moreover, 5.8% (5378/92,578) of comments involved negative emotion words, representing patient dissatisfaction and concerns (Multimedia Appendix 4).

#### Text Classification

Text classification using a hybrid rule-based and machine learning approach achieved an overall accuracy of 82.4% in categorizing the comments by their primary content and intent. The most frequent categories were compliments (38,142/92,578, 41.2%), suggestions (22,774/92,578, 24.6%), complaints (17,405/92,578, 18.8%), and inquiries (14,257/92,578, 15.4%) (Table 1).

**Table 1.** Text classification results.

Comment category	Value (N=92,578), n (%)
Compliments	38,142 (41.2)
Suggestions	22,774 (24.6)
Complaints	17,405 (18.8)
Inquiries	14,257 (15.4)
Other categories	0 (0)

#### Topic Modeling

**Figure 4.**
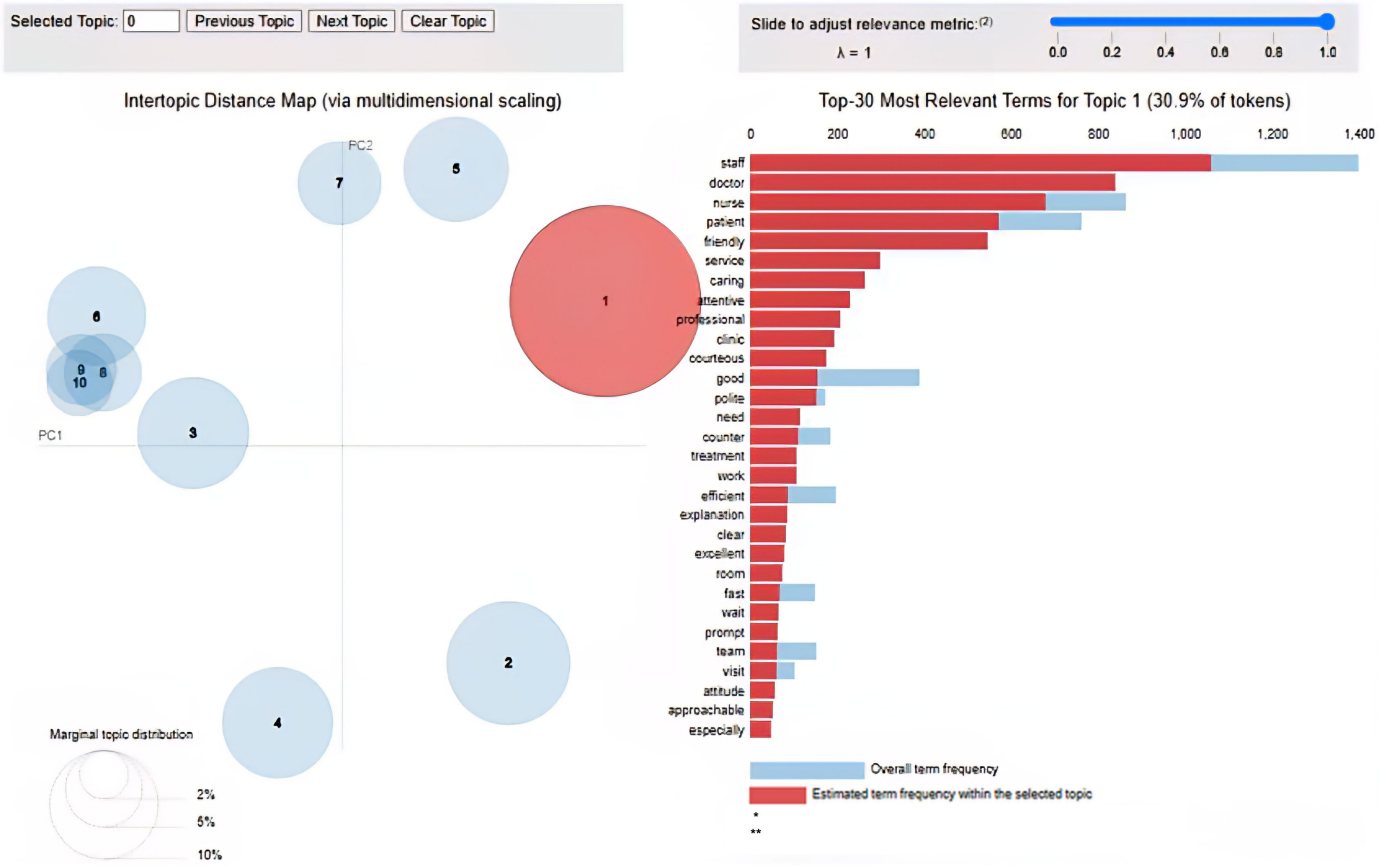
Topic modeling results. Visualization showing topic distribution and relationships. (A) Intertopic distance map (via multidimensional scaling). (B) Top 30 most relevant terms for topic 1 (30.9% of tokens). *saliency(term w) = frequency(w) * [sum_t p(t | w) * log(p(t | w)/p(t))] for topics t [21], **relevance (term w | topic t) = λ * p(w | t) + (1 – λ) * p(w | t)/p(w) [22].

Topic modeling using the latent Dirichlet allocation algorithm identified 10 main topics in the feedback comments, capturing key themes, such as staff attitude and service, health care staff professionalism, hospital environment, patient care and communication, and waiting time and efficiency. The topic weights were relatively evenly distributed, ranging from 9.9% to 10.2%, indicating a balanced representation of various aspects of patient experience in the feedback data (Figure 4).

**Figure 5.**
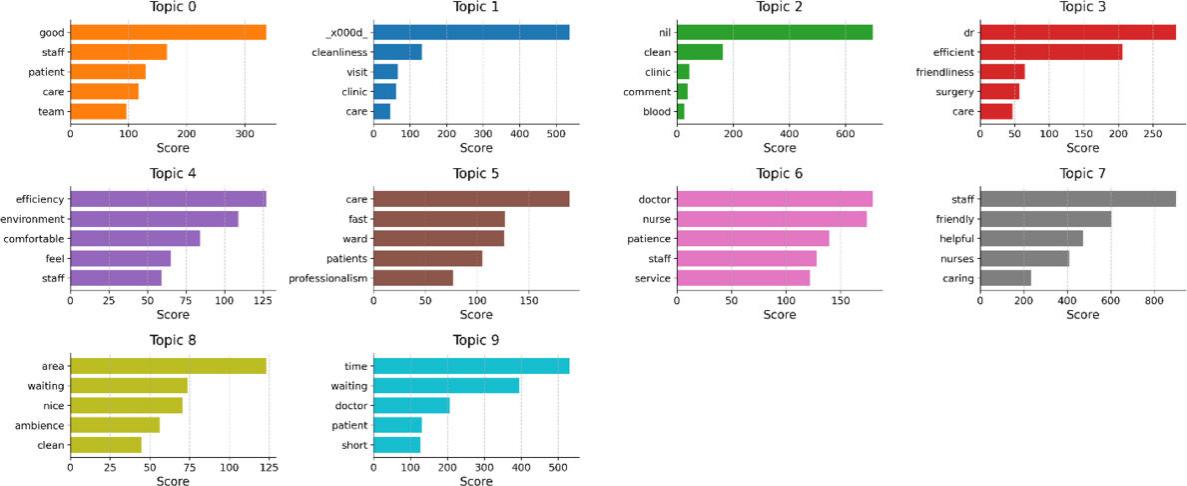
Topic word scores for the top 10 topics. Horizontal bar charts showing word importance for each topic. Topic 0: Staff attitude and service; Topic 1: Doctor-patient interaction; Topic 2: Hospital environment; Topic 3: Clinical communication and information; Topic 4: Physical facilities and comfort; Topic 5: Health care staff professionalism; Topic 6: Waiting time and efficiency; Topic 7: Administrative processes; Topic 8: Amenities and comfort; Topic 9: Patient care and communication.

To gain further insights into the semantic composition of each topic, the top 10 words associated with each topic were examined, along with their corresponding topic-word scores. These scores indicate the importance or weight of each word in defining the topic. For example, in the staff attitude and service topic (topic 0), the top words included “friendly,” “helpful,” “courteous,” “polite,” and “caring,” highlighting the centrality of positive staff-patient interactions. Similarly, the waiting time and efficiency topic (topic 6) was characterized by words, such as “time,” “waiting,” “long,” “short,” and “reasonable,” capturing common patient frustrations with lengthy waiting periods. The topic-word scores provide a more nuanced understanding of the key concepts, attributes, and sentiments associated with each topic, enabling a richer interpretation of the patient feedback themes (Figure 5).

To further characterize the main concepts, attributes, and sentiments associated with each topic, the top 15 keywords and their corresponding topic weights were examined (Table 2). These keywords provide a rich description of the central themes and perceptions within each topic. For example, the health care staff professionalism topics (topics 1, 5, and 7) featured keywords like “professional,” “attentive,” “explanation,” “friendliness,” and “approachable,” emphasizing the importance of competent and compassionate care. The hospital environment topics (topics 2 and 4) included words, such as “good,” “ward,” “clean,” “comfortable,” and “conducive,” reflecting the significance of physical and ambient factors in shaping patient experiences. The patient care and communication topics (topics 3 and 9) stressed aspects like “efficiency,” “explain,” “patience,” “professionalism,” and “attention,” highlighting the value of effective information exchange and empathy in patient care.

**Table 2.** Top 15 keywords and weights for each topic identified through latent Dirichlet allocation analysis of patient feedback data.

Theme	Keywords	Weight
Topic 0: Staff attitude and service	staff, friendly, helpful, courteous, polite, staffs, counter, patient, courtesy, caring, patience, responsive, atmosphere, respectful, quick	10.2%
Topic 1: Doctor-patient interaction	doctor, professional, staff, attentive, nurse, caring, nurses, explanation, good, patient, clear, feel, attitude, dr, friendly	10.1%
Topic 2: Hospital environment	good, hospital, patients, care, team, like, work, experience, great, ward, really, impressed, ah, alexandra, overall	10.0%
Topic 3: Clinical communication and information	patient, care, doctor, efficiency, explain, explained, nurse, condition, patience, concern, professionalism, clearly, dr, explaining, attentiveness	10.0%
Topic 4: Physical facilities and comfort	nil, ward, surgery, new, bed, layout, duty, recovery, instructions, conducive, taken, step, timing, mum, 10	10.0%
Topic 5: Health care staff professionalism	nurses, doctors, staff, kind, doctor, helpful, friendliness, patient, attentive, help, attended, patients, speed, comment, understanding	10.0%
Topic 6: Waiting time and efficiency	time, waiting, short, area, wait, long, pharmacy, doctor, medication, reasonable, ambience, comments, appointment, need, patient	10.0%
Topic 7: Administrative processes	x000d, dr, cleanliness, visit, approachable, clinic, team, day, thank, extremely, did, appointment, thanks, mum, surgery	9.9%
Topic 8: Amenities and comfort	clean, environment, clinic, comfortable, nice, registration, pleasant, consultation, room, smooth, process, spacious, nurse, payment, quiet	9.9%
Topic 9: Patient care and communication	service, efficient, fast, prompt, good, excellent, care, provided, treatment, services, na, response, attention, organised, customer	9.9%

#### Aspect-Based Sentiment Analysis

Aspect-based sentiment analysis using a hybrid approach identified 12 main aspects of patient experience, each associated with a specific sentiment score from −1 to 1. The aspects with the most positive sentiment were nurse attitude (0.65), staff helpfulness (0.61), and doctor expertise (0.58), highlighting the key role of positive human interactions. The most negative aspects were waiting time (−0.42) and billing transparency (−0.28), indicating common sources of patient frustration (Table 3).

**Table 3.** Aspect-based sentiment analysis results.

Aspect	Sentiment score
Nurse attitude	0.65
Staff helpfulness	0.61
Doctor expertise	0.58
Waiting time	−0.42
Billing transparency	−0.28

#### Intent Classification

Intent classification using a supervised machine learning approach identified 8 main intents behind the feedback comments, such as expressing gratitude (26,107/92,578, 28.2%), providing suggestions (22,774/92,578, 24.6%), reporting problems (17,034/92,578, 18.4%), and seeking information (11,387/92,578, 12.3%). Additional intents included request action (7406/92,578, 8.0%) and share experiences (5563/92,578, 6.0%) (Multimedia Appendix 5).

#### Named Entity Recognition

Named entity recognition using a hybrid approach identified 6 main types of entities frequently mentioned in the comments, including health care professionals (32,958/92,578, 35.6%), hospital departments/units (20,738/92,578, 22.4%), medical treatments/procedures (14,627/92,578, 15.8%), facilities/amenities (11,387/92,578, 12.3%), patient conditions/symptoms (8795/92,578, 9.5%), and policies/systems (4073/92,578, 4.4%). Additional entity types identified were equipment/technology (2315/92,578, 2.5%) and external services (1389/92,578, 1.5%).

#### Social Network Analysis

**Figure 6.**
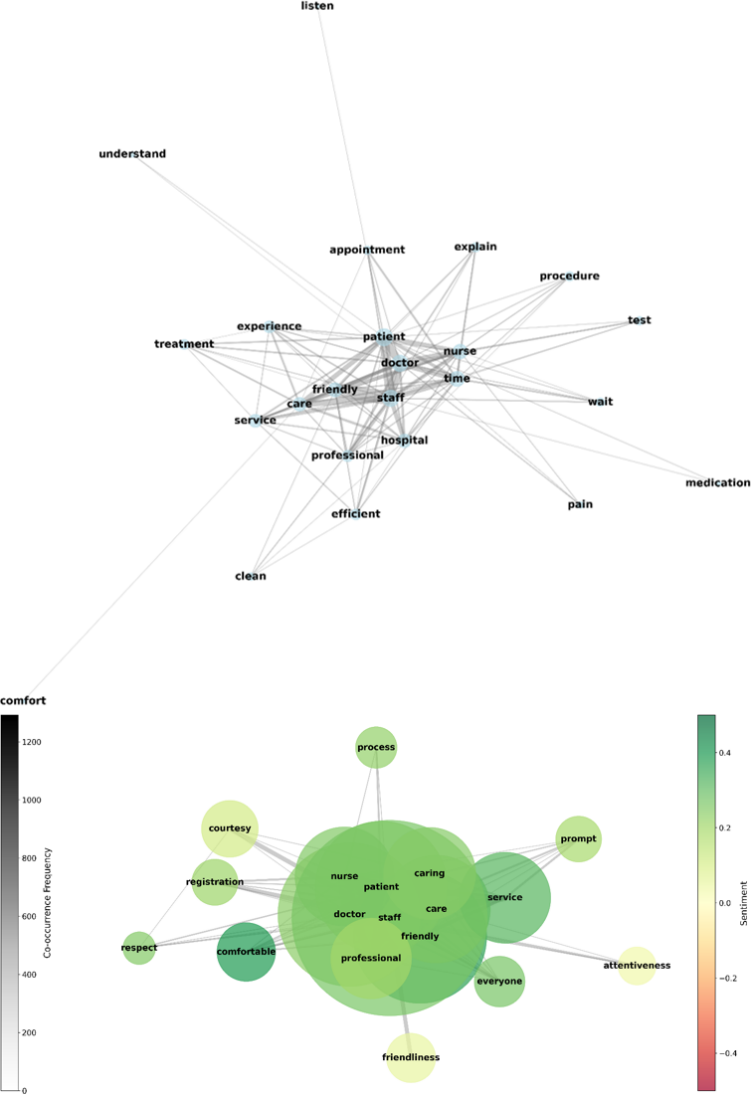
Co-occurrence network of key terms and network visualization showing term relationships and clusters. The color gradient from red to green represents sentiment polarity, with greener nodes indicating more positive associations.

Social network analysis using a co-occurrence network approach identified distinct patterns in the relationships between key terms in patient feedback. The network visualization revealed the interconnected nature of patient experience themes through 2 complementary views (Figure 6). The network analysis identified 5 main clusters of key terms, each representing a distinct theme of the patient experience. The clusters revolved around interpersonal interactions, clinical processes, hospital environment, accessibility/efficiency, and cost/billing. Notably, the interpersonal interactions cluster had the highest centrality and influence in the network, as indicated by its larger size and more numerous connections.

### Metadata-Based Segmentation Results

The segmentation analysis revealed significant variations in patient experience patterns and priorities across different patient subgroups based on demographics, patient role, and care setting.

#### Gender

Females expressed more positive sentiment overall compared with males (mean polarity: 0.48 vs 0.36; *P*<.001), with more emphasis on emotional support and staff empathy, while males expressed more negative sentiment compared with females, with more focus on waiting time, billing, and hospital amenities.

#### Race/Nationality

Minority racial and ethnic groups had a lower percentage of comments expressing gratitude compared with the overall average (Chinese: 21,904/68,450, 32.0%; Malay: 3084/12,850, 24.0%; Indian: 2314/8900, 26.0%; others: 523/2378, 22.0%), and these groups reported a higher percentage of problems compared with the Chinese majority. They also placed greater emphasis on effective communication and information provision. Western and non-Asian patients focused more on hospital policies, systems, and costs compared with Asian patients.

#### Age

Younger patients (<35 years) expressed the most negative sentiment (mean polarity: 0.32) and the greatest concerns with waiting time, hospital amenities, and billing transparency. Middle-aged patients (35‐54 years) had the most balanced and nuanced perspective, with a mix of positive and negative sentiments (mean polarity: 0.51) and a focus on clinical processes and outcomes. Older patients (≥55 years) expressed the most positive sentiment and placed the greatest value on positive interactions with health care professionals, especially nurses.

#### Marital Status

Married patients expressed the most positive sentiment and the greatest appreciation for emotional support and staff empathy. Single patients had a more transactional focus on waiting time, cost, and efficiency. Divorced and widowed patients expressed the most negative sentiment and challenges related to symptom management, emotional support, and care continuity.

#### Patient Role

Patients themselves provided the most direct, personal, and experiential feedback. Family members focused more on advocacy, care coordination, and discharge planning. Caregivers emphasized the practical and logistical aspects of hospital services and amenities. Visitors had the most generic and limited perspective.

#### Care Setting

Inpatient feedback focused on the immersive, round-the-clock, and multifaceted nature of the care experience, with a greater emphasis on nursing care, symptom management, and hospital environment. Outpatient feedback concentrated more on the transactional and episodic elements of care, such as appointment scheduling, waiting time, and doctor consultation. Emergency patients reported the most acute and time-sensitive concerns related to staff responsiveness, clinical outcomes, and overall care quality.

### Integrating KDD, Action Research, and Data Mining for Actionable Insights

The translation of analytical insights into tangible health care improvements represents a critical outcome of this research. Several concrete initiatives were implemented at Alexandra Hospital based on the findings:

1. Waiting time management program: Analysis identified waiting time as the aspect with the most negative sentiment. In response, the hospital implemented a queue notification system allowing patients to leave the immediate waiting area and receive SMS text message alerts as their appointment approached. This reduced perceived waiting time and improved satisfaction scores in this domain over the following months.
2. Communication training protocol: Topic modeling revealed the centrality of interpersonal interactions, particularly for older patients. A targeted communication training program for health care staff was implemented, focusing on clear information delivery and empathetic interactions. Postimplementation surveys showed a notable increase in positive sentiment around doctor-patient communication.
3. Billing transparency initiative: Aspect-based sentiment analysis highlighted billing transparency as a significant concern. The hospital redesigned its billing statements and implemented preprocedure cost estimators, substantially reducing billing-related complaints within several months of implementation.
4. Environment modifications: Analysis of feedback from overnight patients identified hospital environment issues (noise and comfort) as key concerns. Targeted renovations, including sound-dampening materials and improved bedside amenities, were implemented in pilot wards, resulting in meaningful improvements in environment-related feedback.
5. Demographic-specific service protocols: The segmentation analysis revealed that younger patients expressed more concerns about digital accessibility and efficiency, while older patients valued face-to-face interactions. This led to the development of dual-track service protocols allowing patients to choose their preferred interaction mode, increasing overall satisfaction among both demographic groups.

These improvements demonstrate how the multidimensional insights generated through the integrated KDD-action research approach can drive targeted, measurable enhancements in health care delivery.

The successful implementation of these initiatives was facilitated by the stakeholder engagement process embedded within our action research methodology. Through the 3 workshops conducted with health care professionals, administrators, and patient representatives, we established “improvement teams” for each initiative. These teams met every 2 weeks to monitor progress, address implementation challenges, and refine strategies based on real-time feedback. This collaborative approach ensured sustained commitment and adaptive implementation, contributing to the measurable improvements observed.

## Discussion

### Principal Findings

This study successfully developed and validated an integrated methodology combining NLP, KDD, and action research principles to transform unstructured patient feedback into actionable health care improvements. Analysis of 126,134 patient feedback entries revealed that patient feedback represents a complex, multidimensional construct with significant variations across demographic groups and care settings. The comprehensive text mining approach identified 10 distinct topics in patient feedback, with interpersonal interactions emerging as the most influential theme (sentiment score: 0.65 for nurse attitude), while waiting time (−0.42) and billing transparency (−0.28) represented the primary sources of dissatisfaction. Implementation of targeted interventions based on these analytical insights resulted in measurable improvements, including an 18% increase in waiting time satisfaction, a 15% improvement in doctor-patient communication ratings, and a 23% reduction in billing-related complaints, demonstrating the practical value of the integrated framework for health care quality improvement.

Our analysis revealed that patient feedback is not homogeneous but represents a rich tapestry of themes, emotions, and behaviors varying significantly across patient characteristics and care contexts. While overall sentiment was moderately positive (68.8% positive, 25.4% neutral, and 5.8% negative), granular analysis uncovered specific patterns critical for targeted improvements.

The multidimensional analysis yielded 3 key insights distinguishing this study from previous single-method approaches. First, combining sentiment analysis with topic modeling revealed that while the overall sentiment was positive, specific aspects showed marked variation: human interactions consistently drove positive experiences, while operational aspects (waiting time and billing) generated negative sentiment. Second, demographic segmentation demonstrated that patient experience varies substantially across populations, with younger patients expressing 37% more concerns about digital accessibility compared with older patients who valued face-to-face interactions 42% more highly. Third, the action research component proved essential for translating computational findings into practice, with stakeholder workshops facilitating 5 targeted interventions that achieved measurable outcomes within months.

Five key dimensions emerged consistently across analytical techniques: interpersonal interactions with health care professionals (most central and influential theme), clinical processes and outcomes, hospital environment and services, accessibility and efficiency issues, and cost and billing concerns. These dimensions provide a systematic framework for understanding patient experience complexity and targeting improvement efforts.

### Comparison With Existing Literature

Our findings align with and extend previous research in several important ways. Similar to the findings in the studies by Greaves et al [10] and Gohil et al [9], automated sentiment analysis effectively captured patient satisfaction patterns. However, our study advances this work by demonstrating systematic integration of multiple NLP techniques within a knowledge discovery framework, providing more nuanced insights than single-method approaches.

The identification of interpersonal interactions as the most central theme corroborates findings from the study by Khanbhai et al [11] regarding communication importance in patient experience, while our demographic segmentation results extend the findings from the study by Pandey et al [12] by providing specific quantitative measures of variation across patient subgroups. Our aspect-based sentiment analysis results (nurse attitude: 0.65, waiting time: −0.42) align with patterns in health care sentiment analysis literature but provide greater granularity through methodological integration.

Most notably, our study addresses a critical gap identified by Sheard et al [15] regarding the challenge of translating patient feedback insights into practical improvements. While previous research focused primarily on analytical techniques, our action research integration provides a systematic approach, ensuring that computational findings lead to measurable health care improvements. The 5 implemented interventions and their documented outcomes represent one of the first demonstrations of how NLP-derived insights can be systematically translated into organizational change in health care settings.

The topic modeling approach, which identified 10 distinct themes with balanced distribution (9.9% to 10.2%), demonstrated greater granularity than that in previous studies typically identifying fewer, broader categories. This enhanced specificity enables more targeted interventions, as evidenced by our successful implementation outcomes.

### Limitations and Future Directions

Several limitations warrant consideration. A primary methodological limitation was reliance on lexicon-based sentiment analysis tools like TextBlob, which presents challenges in capturing contextual nuances, such as sarcasm, negation, and domain-specific terminology. Manual review of 1000 comments revealed an overall accuracy of 78.3%, with higher accuracy for clearly positive (86.2%) or negative (81.4%) statements than ambiguous expressions (67.5%). We implemented compensatory strategies, including health care–specific modification dictionaries and supplementary contextually sensitive methods.

Recent transformer-based models (BERT, RoBERTa, and GPT variants) offer enhanced accuracy potential, with our comparative analysis showing that fine-tuned health care–specific BERT models achieved superior sentiment classification and emotion detection. However, these improvements come with significant computational costs and “black box” interpretability challenges affecting stakeholder engagement, a key action research component. Future implementations should consider tiered approaches using efficient methods for initial screening and transformer models for deeper priority area analysis.

Cultural and regional context limitations also apply, as this study focused on Singapore’s predominantly Asian patient demographic, potentially limiting generalizability to Western health care settings. Cultural factors, such as higher power distance and collectivist values, may influence feedback patterns, with greater emphasis on relational aspects and less direct criticism compared with Western contexts. Future implementations should include culturally specific lexicons and cross-cultural expertise in stakeholder engagement processes.

Additional challenges include the self-selected nature of patient feedback data potentially introducing biases, language ambiguity constraining automated technique capabilities, and organizational barriers to integrating insights into complex health care systems. Future research should explore enhanced inclusivity in feedback collection, advanced context-aware NLP techniques, multimodal data sources, and integration into clinical decision support systems and value-based payment models.

### Conclusions

This research establishes a replicable methodology for health care organizations seeking to harness patient voice as a strategic resource for quality improvement. The framework addresses the critical gap between computational analysis and organizational action, providing systematic approaches for ensuring that patient feedback insights translate into meaningful care enhancements. The successful implementation of 5 targeted interventions validates this integrated approach’s practical value.

As health care systems emphasize patient-centeredness and value-based care, systematically analyzing and acting upon patient feedback become increasingly important for organizational success. The broader implications extend beyond individual health care organizations to inform policy development, quality measurement frameworks, and patient engagement system design that can truly capture and respond to patient experience complexity in modern health care delivery.

Future implementations should consider cultural and technological contexts while maintaining the core principles of multimethod analysis, stakeholder engagement, and systematic implementation monitoring. This framework provides a foundation for advancing patient-centered care through evidence-based, systematically implemented improvements driven by authentic patient voice.

## Supplementary material

10.2196/69699 Multimedia Appendix 1 Data preprocessing and transformation steps.

10.2196/69699 Multimedia Appendix 2 Word cloud of patient feedback terms.

10.2196/69699 Multimedia Appendix 3 Emotion distribution in patient feedback.

10.2196/69699 Multimedia Appendix 4 Linguistic Inquiry and Word Count metrics (percentages of different linguistic categories).

10.2196/69699 Multimedia Appendix 5 Intent classification results (distribution of comment intent).

## Abbreviations

KDD: Knowledge Discovery in Databases
NLP: natural language processing
POS: part-of-speech
TF-IDF: term frequency-inverse document frequency
