# Supplementary material for: Transforming Patient Feedback Into Actionable Insights Through Natural Language Processing: Knowledge Discovery and Action Research Study
Source: JMIR Form Res. 2025 Aug 26;9:e69699. doi: 10.2196/69699 (PMC12381215; doi:10.2196/69699)
Supplement: Multimedia Appendix 1 [file formative-v9-e69699-s001.docx]

**Section S1: Data preprocessing steps in detail**

The pre-processing workflow consisted of the following sequential steps:

1. Text Cleaning: Raw text was first cleaned by removing HTML tags, URLs, email addresses, and special characters using regular expressions. Non-English characters were retained to preserve multilingual content reflective of Singapore's diverse population.
2. Tokenization: Text was segmented into individual tokens using NLTK's word_tokenize function with the Punkt tokenizer model.
3. Case Normalization: All tokens were converted to lowercase to ensure consistent processing while preserving abbreviations and acronyms common in healthcare contexts.
4. Stopword Removal: A customized stopword list was created by combining NLTK's English stop words with domain-specific terms identified through manual review of 500 randomly selected comments. Healthcare-specific stopwords included common filler words like 'hospital', 'doctor', 'nurse' when used without descriptive context.
5. Punctuation Handling: Punctuation marks were removed except for periods in abbreviations (e.g., 'Dr.', 'etc.') and hyphens in compound medical terms.
6. Lemmatization: Words were reduced to their base forms using NLTK's WordNetLemmatizer with part-of-speech tagging to ensure accurate lemmatization (e.g., 'caring' → 'care', 'better' → 'good').
7. Filtering: Tokens shorter than 2 characters or longer than 20 characters were removed, along with purely numeric strings.

**Section S2. Data transformation steps in detail**

1. TF-IDF Vectorization: Term Frequency-Inverse Document Frequency matrices were constructed using scikit-learn's TfidfVectorizer with the following parameters: max_features=5000, min_df=5, max_df=0.8, ngram_range=(1,2). This captured both unigrams and bigrams while filtering rare and overly common terms.
2. N-gram Extraction: Bigrams and trigrams were extracted using NLTK's ngrams function with frequency thresholds of minimum 10 occurrences for bigrams and 5 occurrences for trigrams. Collocations were identified using pointwise mutual information (PMI) scoring with a threshold of 3.0.
3. Part-of-Speech Tagging: POS tagging was performed using NLTK's averaged perceptron tagger, with focus on extracting adjectives (describing qualities), nouns (entities), and verbs (actions). Healthcare-specific POS patterns were identified, such as adjective-noun pairs describing staff attributes.
4. Feature Engineering: Additional features were created including:
   - Sentiment polarity scores using TextBlob
   - Text length metrics (character count, word count, sentence count)
   - Readability scores using the Flesch Reading Ease formula

Emotion intensity scores using the NRC Emotion Lexicon
