# Supplementary figures and images for: Transforming Patient Feedback Into Actionable Insights Through Natural Language Processing: Knowledge Discovery and Action Research Study
Source: JMIR Form Res. 2025 Aug 26;9:e69699. doi: 10.2196/69699 (PMC12381215; doi:10.2196/69699)

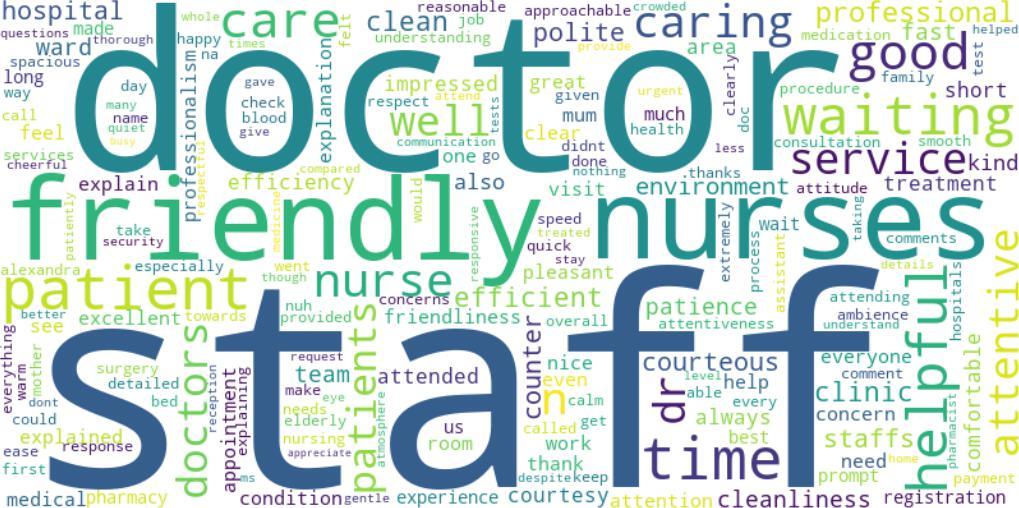

Supplement: Multimedia Appendix 2 [file formative-v9-e69699-s002.png]

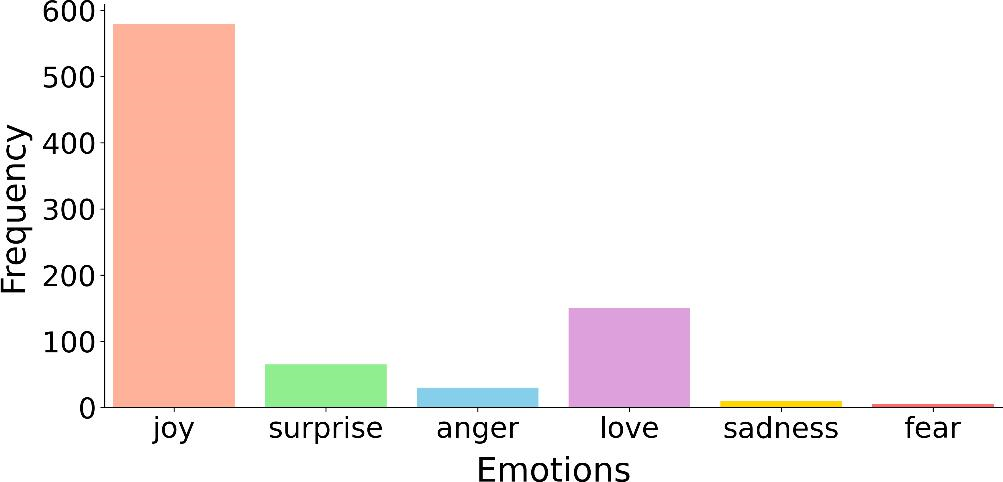

Supplement: Multimedia Appendix 3 [file formative-v9-e69699-s003.png]

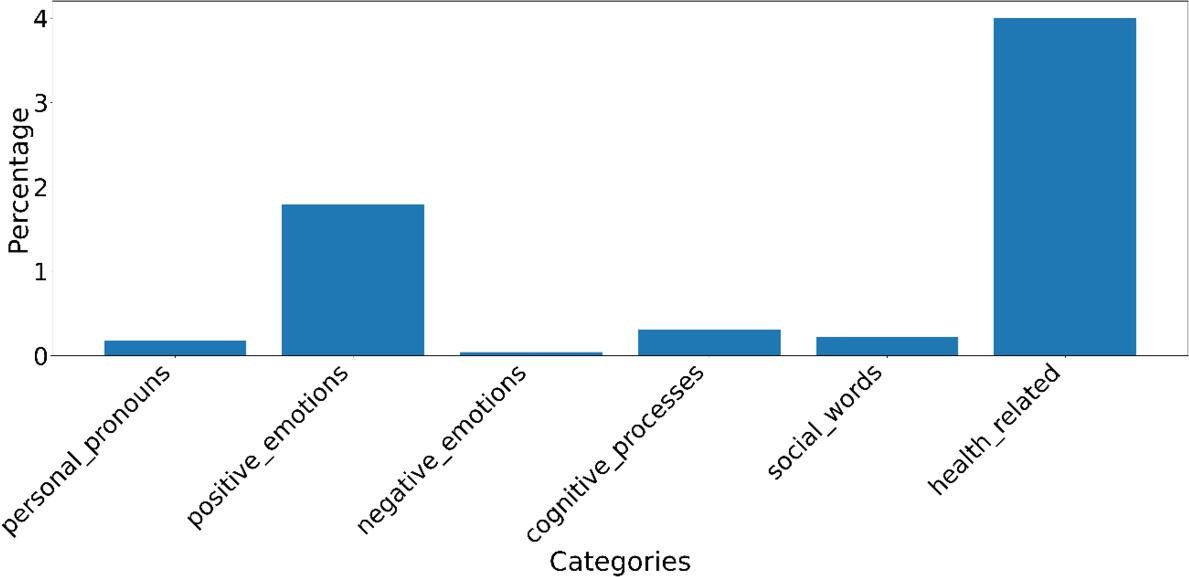

Supplement: Multimedia Appendix 4 [file formative-v9-e69699-s004.png]

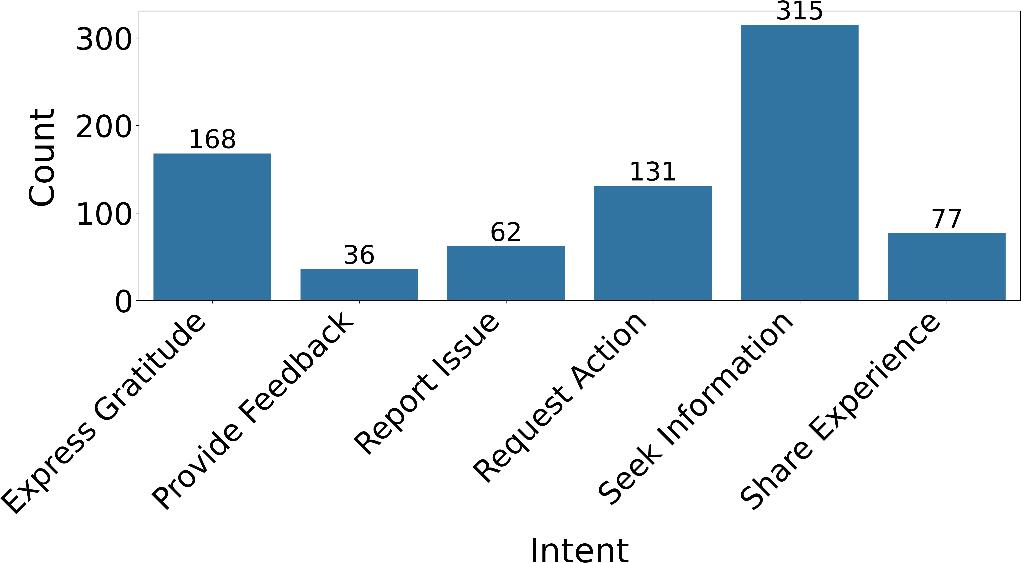

Supplement: Multimedia Appendix 5 [file formative-v9-e69699-s005.png]
